# Correlations between gut microbiota community structures of Tibetans and geography

**DOI:** 10.1038/s41598-017-17194-4

**Published:** 2017-12-05

**Authors:** Daoliang Lan, Wenhui Ji, Baoshan Lin, Yabing Chen, Cai Huang, Xianrong Xiong, Mei Fu, Tserang Donko Mipam, Yi Ai, Bo Zeng, Ying Li, Zhixin Cai, Jiangjiang Zhu, Dawei Zhang, Jian Li

**Affiliations:** 1 Institute of Qinghai-Tibetan Plateau, Southwest University for Nationalities, Chengdu, 610041 People’s Republic of China; 2 College of Life Science and Technology, Southwest University for Nationalities, Chengdu, 610041 People’s Republic of China; 3 Animal Disease Prevention and Control Center of Aba Tibetan and Qiang Autonomous Prefecture, Sichuan Province, 624000 People’s Republic of China; 4 Farm Animal Genetic Resources Exploration and Innovation Key Laboratory of Sichuan Province, Sichuan Agricultural University, Chengdu, 611130 People’s Republic of China

## Abstract

Microbial communities of human gut directly influence health and bear adaptive potential to different geography environment and lifestyles. However, knowledge about the influences of altitude and geography on the gut microbiota of Tibetans is currently limited. In this study, fecal microbiota from 208 Tibetans across six different locations were analyzed by MiSeq sequencing; these locations included Gannan, Gangcha, Tianzhu, Hongyuan, Lhasa and Nagqu, with altitudes above sea level ranging from 2800 m to 4500 m across the Tibetan plateau. Significant differences were observed in microbial diversity and richness in different locations. At the phylum level, gut populations of Tibetans comprised Bacteroidetes (60.00%), Firmicutes (29.04%), Proteobacteria (5.40%), and Actinobacteria (3.85%) and were marked by a low ratio (0.48) of Firmicutes to Bacteroidetes. Analysis based on operational taxonomic unit level revealed that core microbiotas included *Prevotella*, *Faecalibacterium*, and *Blautia*, whereas *Prevotella* predominated all locations, except Gangcha. Four community state types were detected in all samples, and they mainly belong to *Prevotella*, *Bacteroides*, and *Ruminococcaceae*. Principal component analysis and related correspondence analysis results revealed that bacterial profiles in Tibetan guts varied significantly with increasing altitude, BMI, and age, and facultative anaerobes were rich in Tibetan guts. Gut microbiota may play important roles in regulating high-altitude and geographical adaptations.

## Introduction

The Tibetan Plateau is considered the third pole and is one of the places on earth with most extreme living conditions^1^. This place is characterized by low oxygen levels, high radiation, and shortage of supplies^1,2^. These conditions are formidable physiological challenges for people or animals living in this high-altitude plateau^3^. However, indigenous people of Tibet occupied the harsh place for more than 25,000 years and established a major civilization in the Himalayan and other regions^4–6^. These Tibetans serve as good examples of successful high-altitude adaptation^3^. Tibetans are also good examples for exploring mechanisms of high-altitude adaptation. Numerous works were conducted to uncover molecular signatures of high-altitude adaptation across a wide range of geographic locations using genome-wide analyses^3,7–10^. Tibetans also developed unique lifestyles and dietary habits, e.g., meat (beef and mutton), yak butter, milk, and other dairy products are major foods for energy, but minimal vegetables and fruits are consumed^11^.

Gut microbiota can directly influence human health and show adaptive potential to different lifestyles^12^. These gut biomes represent the largest cell pools of the human body, and they are vital for host nutrition, metabolism, pathogen resistance, and immune function^13^. Microbiota structures vary with genetics, mode of delivery, diet, lifestyle, age, medical treatments, and other factors^14,15^. Some studies also centered on correlations between community structures of gut microbiota and geography^16–19^. Children living in rural regions showed higher bacterial diversity and lower Firmicutes/Bacteroidetes (F/B) ratio than those from developed regions^15,16^. Results of gut microbiome survey across three countries revealed that people living in Venezuela, Malawi, and the United States of America exhibited different gut bacterial profiles that correlated with geographic origins and age^20^. However, some studies reported absence of significant structural changes in gut microbiota of individuals from six countries, and effects of body mass index, age, or gender were not observed on gut microbiota structure^21^. Diverse gut compositions of people living in different regions may contribute to complexity of microbiota associated with hosts and further confirm the importance of unveiling gut microbiota diversity to uncover roles of microbiotas in host health and to discover new means of adaptation to different environments^22^.

Genetic differences were revealed by genome-wide analysis or other molecular methods between Tibetans and Han people^3,7–10^. Some studies compared fecal microbiotas between Tibetan and Han populations and Mongolians, suggesting different gut microbiome structures in Tibetans^2,19,20^. Compared with Han populations, Tibetan microbiome was characterized by relative abundance of *Prevotella*, whereas Han stool was enriched with *Bacteroides*^23^. Tibetans living at high altitudes (4800 m) showed microflora enriched with butyrate-producing bacteria in response to harsh environments^23^. Short-chain fatty acids (SCFAs) produced by *Clostridium*, *Desulfovibrio*, *Bacteroides*, *Lactobacillus*, and *Prevotella* can help in decreasing blood pressure and adapting to energy demands and pulmonary hypertension^2,23^. However, no reports were received for uncovering fecal microbiota of Tibetans at a large scale (Tibetan Plateau measures approximately 2,500,000 km^2^). Different geographical locations, lifestyles, farming styles, and frequencies of communication with other places may vary with gut microbiome of Tibetans.

In this study, to determine correlations between gut microbiota community structures of Tibetans and geography, we analyzed feca microbiotas from 208 samples from six regions with altitudes ranging from 2800 m to 4500 m across the Tibetan Plateau and compared phylogenetic diversity and taxonomic relative abundance among these regions.

## Results

### DNA Sequencing and filtering

A total of 17,870,011 raw reads were generated from the MiSeq platform. After filtering low-quality reads, 16,509,385 clean reads were retained with lengths measuring 240–300 bp, and nearly 7.04% of raw data were filtered. Average number of high-quality reads in each sample reached 79,372 and ranging from 12,594 to 125,895 across all samples.

### Microbial diversity in samples from different places in Tibetan Plateau

**Figure 1:**
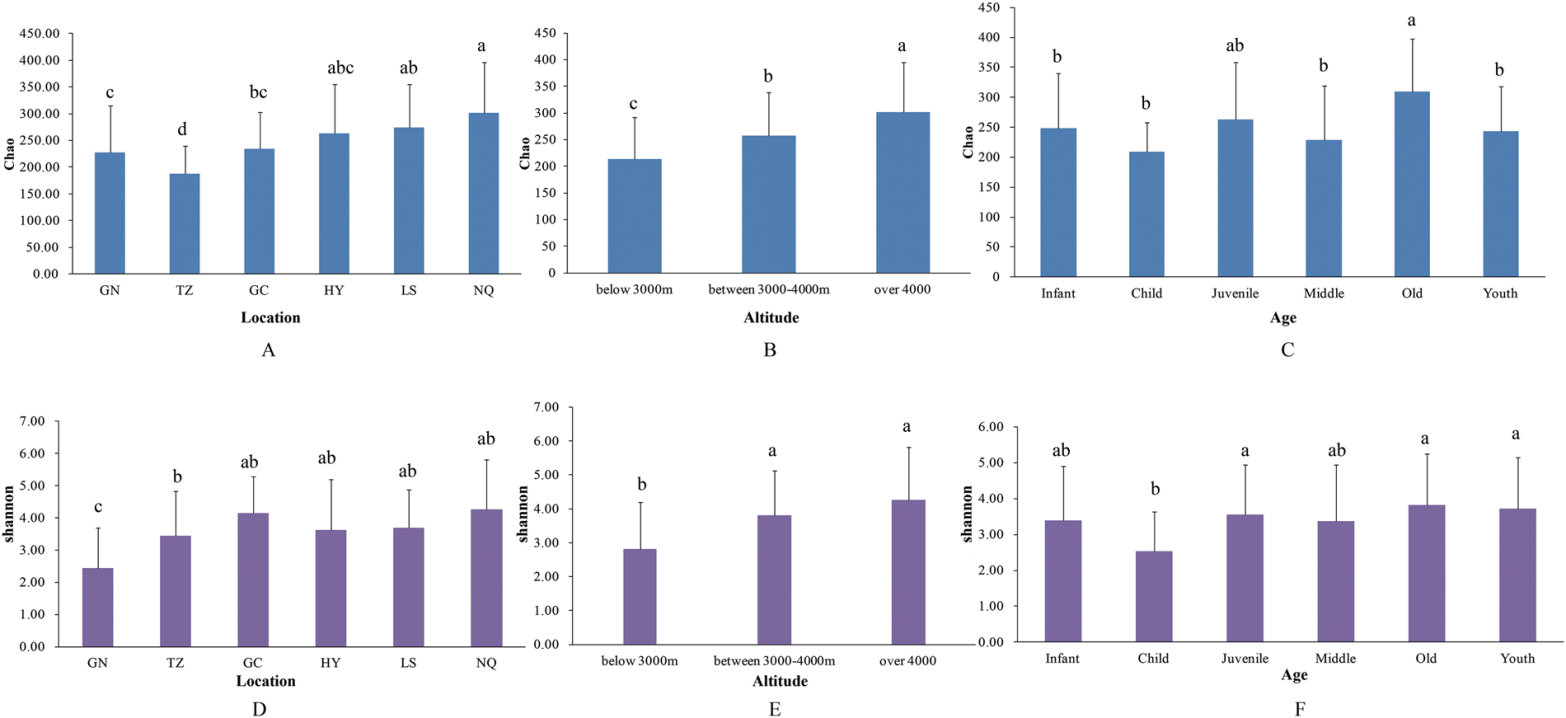
Microbial diversity and richness in samples. (**A**–**C**) Chao index. (**A**) Different locations, (**B**) Altitude, (**C**) Ages; (**D–F**) Shannon index. (**D**) Different locations, (**E**) Altitude, (**F**) Ages. The small ‘abcd’ letters above the bars represent differences between groups, the same letter indicating that the difference is not significant, whereas the difference letter indicating that the difference is significant.

All 16,509,385 high-quality sequences were clustered into operational taxonomic units (OTUs) at 97% sequence similarity using Quantitative Insights Into Microbial Ecology (QIIME) software. A total of 1,544 OTUs were detected (Table S1). Rarefaction curves showed that plateau level was reached in all samples (Figure S1) with Good’s coverage value ranging from 98.94% to 99.84%, revealing that our sequencing depth was adequate to mine microbial community in fecal samples. Microbial diversity (Shannon index)and richness (Chao index)showed significant differences across samples in different locations (Fig. 1). Shannon index was the highest in Hongyuan (HY), Lhasa (LS), and Nagqu (NQ)samples, whereas Chao index was the highest in Gangcha (GC), HY, LS, and NQ samples. Lowest Chao index was 187 in Tianzhu (TZ), whereas lowest Shannon index reached 2.44 in Gannan (GN).

Altitude of locations of GN and TZ measured below 3000 m, whereas that of locations LS, HY, and GC was between 3000 and 4000 m. NQ was the harshest location with average altitude of over 4000 m. Along with rising altitude, bacterial diversity in gut increased from 2.80 in GN and TZ to 4.27 in NQ, whereas richness in gut rose from 212 to 301.

Bacterial diversity and richness also correlated with age. In the old stage, bacterial diversity and richness were the highest, reaching 3.82 and 309, respectively. Bacterial diversity and richness increased with people’s growth. However, no correlation exists between gut microbiomes and BMI (data not shown).

### Beta diversity of gut microbiota among different places in Tibetan Plateau

**Figure 2:**
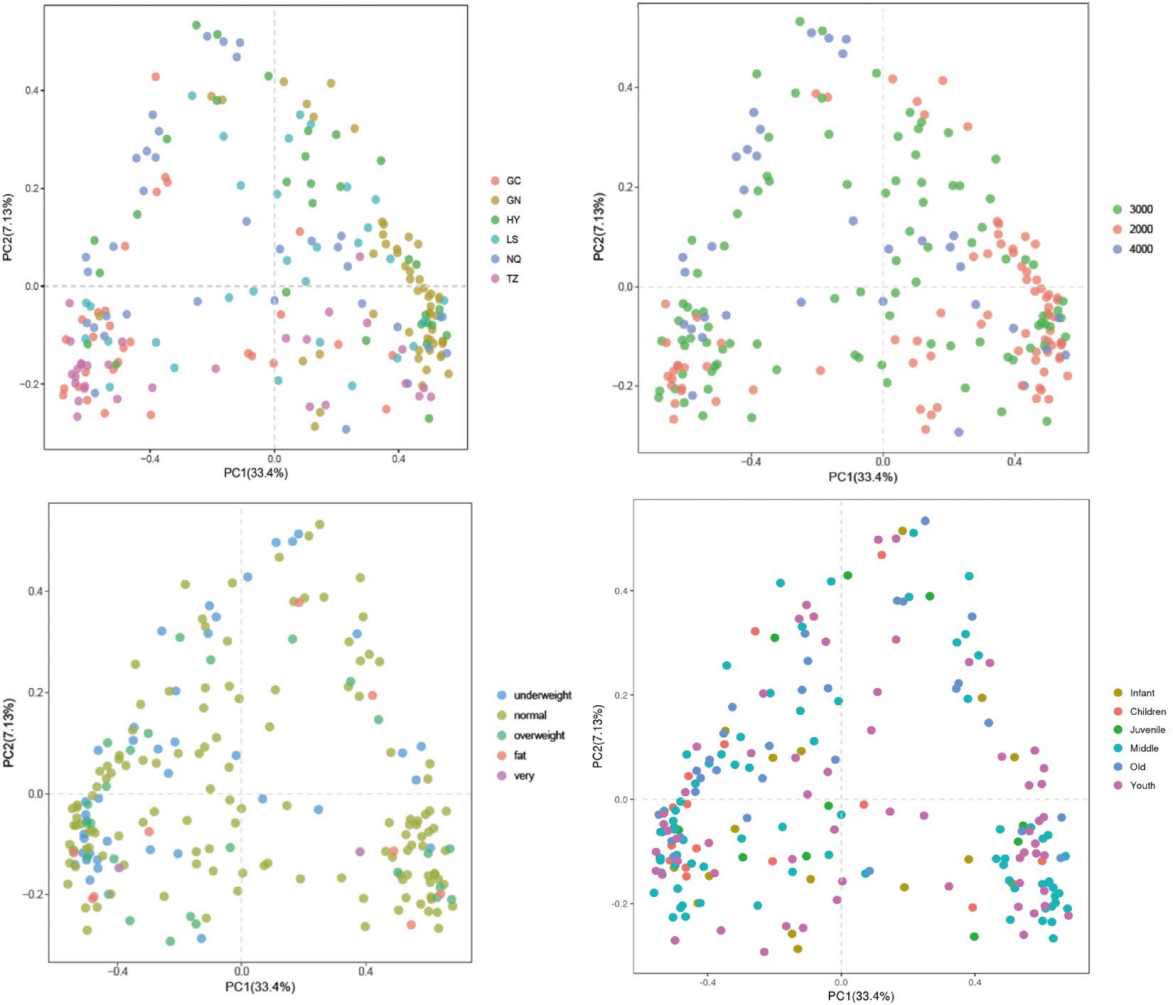
PCA analyses for detecting similarities between different samples. (**A**) Different locations, (**B**) Altitude, (**C**) BMI, (**D**) Ages.

Comparisons were conducted to uncover differences among samples from different places. Principal component analysis (PCA) and cluster analysis suggested that significant differences were observed across samples from different places (p < 0.001) (Fig. 2A). Analysis of Variance (ANOVA) further confirmed that samples were significantly different from other samples at p < 0.05, revealing differences in microbial community between these samples.

Bacterial compositions in guts of Tibetans varied significantly with increasing altitude (Fig. 2B). In terms of BMI, only underweight participants presented different bacterial profiles compared with normal-weight and obese participants. However, no differences were observed among other participants (Fig. 2C). Under growth processes, bacterial communities varied significantly in stages of child and youth; and old and youth (Fig. 2D).

### Bacterial compositions of guts of Tibetans in different places

Nearly all sequences (99.99%) in the dataset were assigned to a bacterial kingdom, but a few reads remained unclassified. Overall, 18 bacterial phyla were recovered from samples; these included Bacteroidetes (60.00%), Firmicutes (29.04%), Proteobacteria (5.40%), and Actinobacteria (3.85%) (Figure S2), which accounted for 90% of total sequences. However, the proportions of the same bacteria in samples from different regions were different at the phylum level. The relative abundance of Bacteroidetes in GN (82.27%) was highest among all groups, followed by HY (66.99%), TZ (54.48%), LS (59.12%), NQ (51.10%) and GC (45.90%). Firmicutes in NQ (40.00%) and GC (41.49%) samples were more abundant than those in HY, LS, TZ, and GN samples. Relative abundance of Firmicutes in GN samples reached11.71%, which is much lower than those of other samples, especially GN samples. Actinobacteria in NQ (3.85%) samples were much lower than those in other samples. F/B ratio totaled 0.48 in all samples. F/B ratios were 0.14, 0.37, 0.46, 0.58, 0.78, and 0.90 in GN, HY, LS, TZ, NQ, and GC populations, respectively.

At the class level, 34 classes were detected in all samples (Figure S3), of which 91.20% were represented by bacteria belonging to classes Bacteroidia, Clostridia, Gammaproteobacteria, and Actinobacteria. Statistics showed that 25 classes varied significantly in all samples (*p* < 0.05). Percentage of reads belonged to Bacteroidia reached 82.27%, 66.99%, 59.11%, 54.48%, 51.09%, and 45.90% in samples from GN, HY, LS, TZ, NQ, and GC, respectively. Relative abundance of Clostridia measured 8.53% in GN samples; this value was the lowest in all samples.

At the family level, 102 families were detected in all samples, of which 75.00% were represented by bacteria belonging to families Prevotellaceae, Ruminococcaceae, Bacteroidaceae, and Lachnospiraceae (Figure S4). At the genus level, nearly all samples were dominated by *Prevotella*, except GC, which is dominated by *Bacteroides* (22.78%) (Figure S5). In GN samples, percentage of *Prevotella* reached 75.71%. In locations HY, LS, NQ, TZ, and GC, percentages of *Prevotella* totaled 44.32%, 41.17%, 33.02%, 31.81%, and 16.15%, respectively. Probiotic *Bifidobacterium* was also present in all samples. Relative abundance of *Bifidobacterium* reached 5.94%, 3.35%, and 2.23% in GC, NQ, and LS samples. However, in GN and HY samples, relative abundance of *Bifidobacterium* was lower than 1%.

### Core and shared bacteria in guts of Tibetans

Core compositions of gut bacteria in 208 samples of Tibetans were detected based on OTUs. Three core OTUs were obtained, and they belonged to *Prevotella* (Prevotellaceae), *Faecalibacterium* (Clostridiaceae), and *Blautia* (Lachnospiraceae) (Table 1). OTU belonging to *Prevotella* was the most abundant.

**Table 1:** Core bacterial compositions in all gut samples.

	GC	GN	HY	LS	NQ	TZ
*Prevotella*	13.26 ± 23.09	60.80 ± 23.68	37.80 ± 31.63	32.97 ± 26.60	24.00 ± 29.72	24.92 ± 33.61
*Faecalibacterium*	5.52 ± 6.33	0.75 ± 0.89	2.70 ± 4.37	3.38 ± 6.22	2.69 ± 2.86	4.67 ± 5.65
*Blautia*	0.29 ± 0.61	0.06 ± 0.12	0.33 ± 0.42	0.12 ± 0.13	0.20 ± 0.24	0.18 ± 0.32

**Figure 3:**
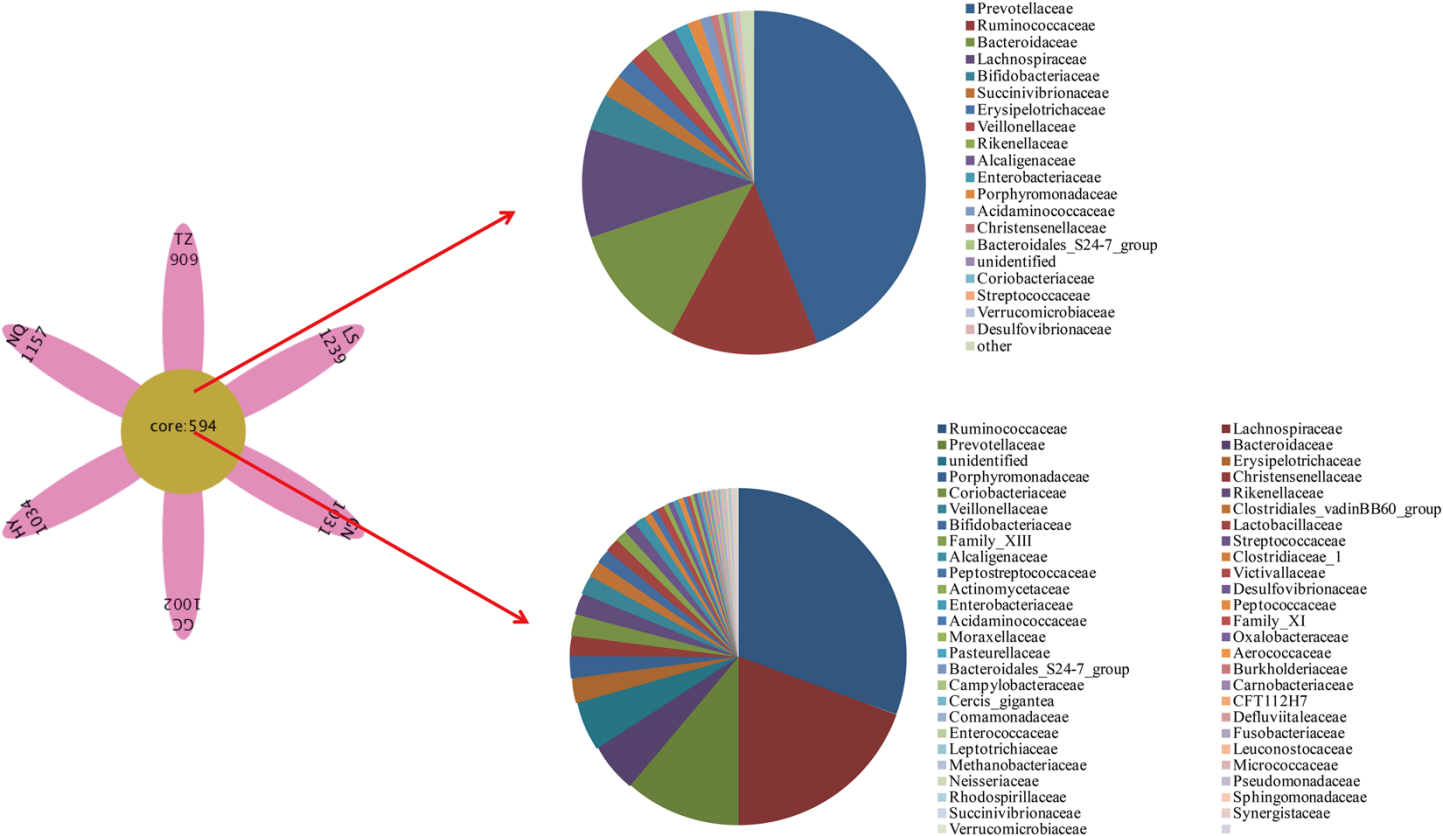
VENN analyses among different locations.

Figure 3 illustrates common OTUs shared by locations GC, GN, HY, LS, NQ, and TZ, with a total of 594 OTUs detected. These 594 OTUs were assigned to 53 different families, and the numbers of OTUs belonging to Ruminococcaceae, Lachnospiraceae, Prevotellaceae, and Bacteroidaceae totaled 182, 115, 66, and 28, respectively. The most abundant family was Prevotellaceae, representing 42.86% of total sequences. Abundance of Ruminococcaceae (13.60%) and Bacteroidaceae (11.62%) was higher than 10% of total sequences.

### Microbial signatures in different samples

VENN analyses showed that some OTUs were unique to some locations, e.g., 909 OTUs in TZ, 1157 in NQ, 1034 in HY, 1002 in GC, 1031 in GN, and 1239 in LS (Fig. 3)

**Figure 4:**
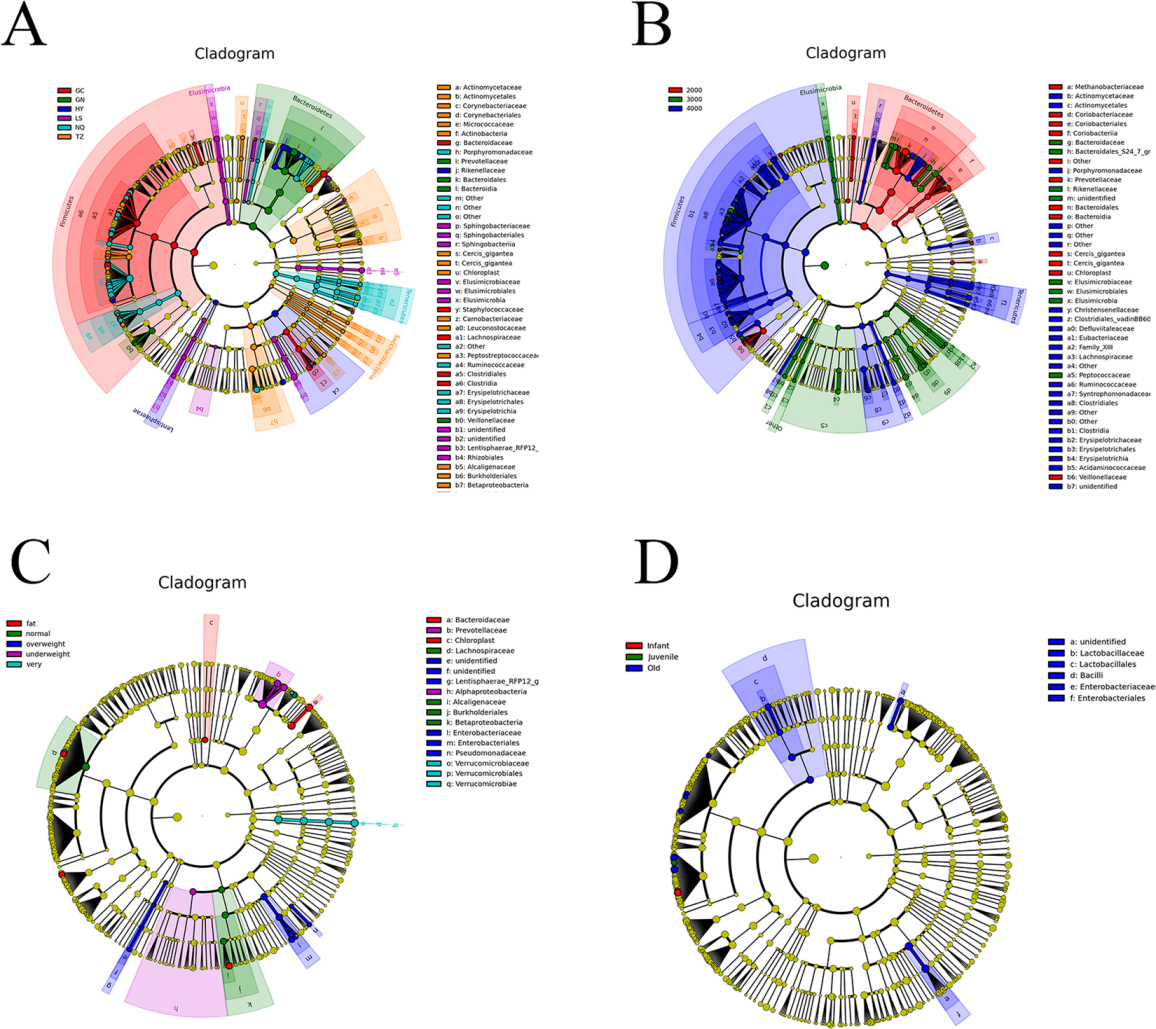
LEfSe conducted based on bacterial community in all samples. (**A**) Different locations, (**B**) Altitude, (**C**) MBI, (**D**) Ages.

Linear discriminant analysis effect size (LEfSe) was further conducted to detect microbial signature in each location. Signature gut microbiota included Prevotellaceae, Bacteroidales, and Veillonellaceae in GN sample; Bacteroidaceae, Staphylococcaceae, Lachnospiraceae, and Clostridiales in GC sample; Micrococcaceae in LS sample; Rikenellaceae in HY sample; Porphyromonadaceae, Ruminococcaceae, and Erysipelotrichaceae in NQ sample; Prevotellaceae in TZ sample; Sphingobacteriaceae, Elusimicrobiaceae, and Rhizobiales in LSsample; and Clostridiaceae in GC sample (Fig. 4A).

Methanobactericeae, Coriobacteriaceae, Prevotellaceae, Bacteroidales, and Veilonellaceae were signature microbiota in guts of participants living in environments under 3000 m. Actinomycetales, Porphyromonadaceae, and Clostridiales were signature microbiota in samples from locations between 3000 and 4000 m (Fig. 4B). Bacteroidaceae, Enterobacteriaceae, and Verrucomicrobiae were signature microbiota in guts of obese participants (Fig. 4C). Only old people presented unique bacterial taxes belonging to Lactobacillales and Enterobacteriales (Fig. 4D).

### Correlations between gut microbiome and age and altitude and body mass index (BMI)

**Figure 5:**
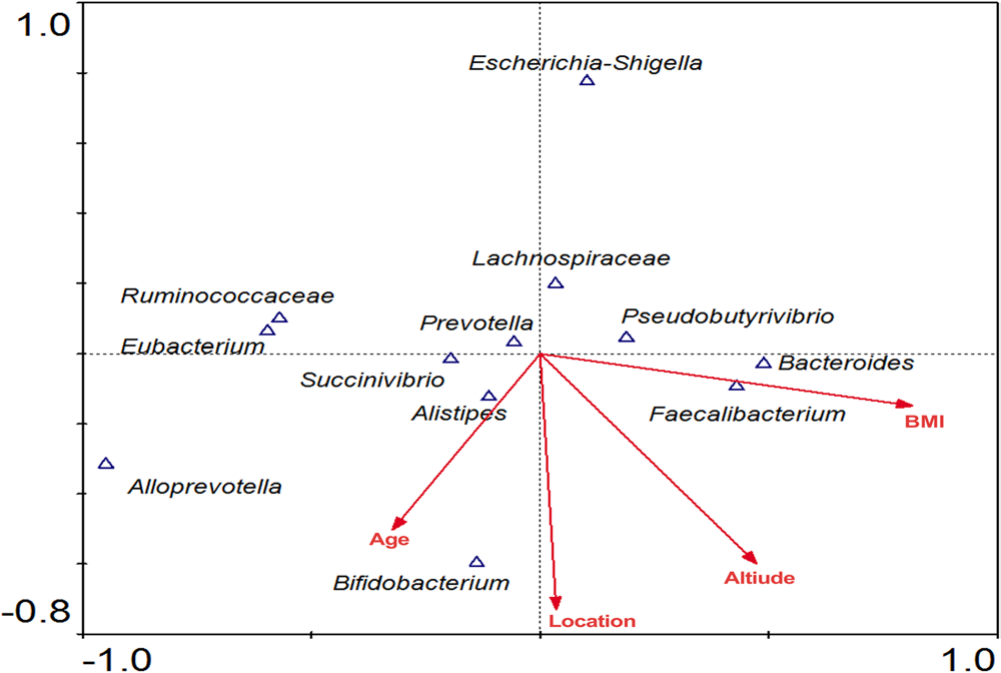
CCA of age, altitude, BMI, and location with community composition at the genus level.

Correlations between bacterial community and locations, age, altitude, and BMI were determined using microbial composition at the genus level (>1%). Results of canonical correspondence analysis (CCA) revealed that different taxes in guts were influenced by age, altitude, and BMI. Ruminococcaceae, Prevotella and Lachnospiraseae were main negative with altitude. Faecalibacterium, Bacteroides and Bifidobacterium was positive with altitude, BMI and age (Fig. 5).

### Community State Types (CSTs) analysis

**Figure 6:**
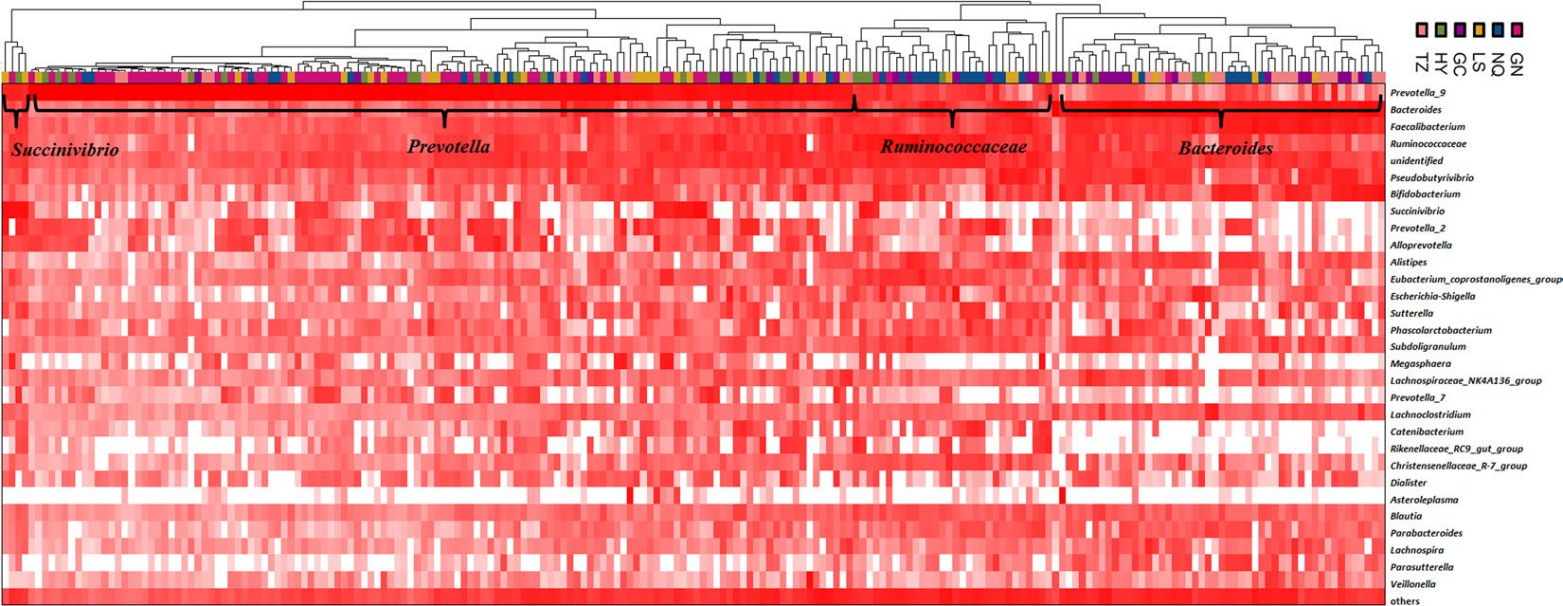
Heat map of complete linkage clustering of samples based on genus composition and abundance in communities.

The CSTs analysis revealed that four CSTs are present in the Tibetans, namely, *Bacteroides*, *Prevotella*, *Ruminococcaceae* and *Succinivibrio* (Fig. 6). *Prevotella* CST, which was present in 124 out of 208 participants, was the most abundant, followed by *Bacteroides* and *Ruminococcaceae* CSTs. In addition, the fourth CST, which belonged to *Succinivibrio*, was detected in two samples from LS, one sample from GN and one sample from HY (Table 2).

**Table 2:** Sample size of four CSTs from different areas.

CSTs	Locations						Total
	GC	LS	NQ	TZ	GN	HY	
*Bacteroides*	16	5	7	17	1	4	50
*Ruminococcaceae*	6	4	13	0	2	5	30
*Prevotella*	8	21	14	13	48	20	124
*Succinivibrio*	—	2	—	—	1	1	4

## Discussion

A survey of gut microbiota of Tibetans from six locations was conducted using the MiSeq sequencing platform. Results revealed that bacterial compositions in guts of Tibetans varied significantly with increasing altitude, BMI, and age. Core microbiota included *Prevotella*, *Faecalibacterium*, and *Blautia*. Four CSTs were detected in all samples.

Host geographic location, lifestyle, diets, and age play important roles in shaping structure of gut microbial communities based on surveys of populations from the United States, Europe, and Korea^15,24^. Bacterial community structure in Tibetans was correlated with factors mentioned above. However, bacterial diversity and richness were not significantly correlated with BMI. Core microbiota comprised *Prevotella*, *Faecalibacterium*, and *Blautia* in Tibetans. *Prevotella* was the dominant genus; this result is consistent with those of other studies. The three genera were common for core OTUs in Chinese and Western populations^2^. Previous studies at functional and metabolic levels indicated that these genera play key roles in synthesizing basic metabolites in human gastrointestinal tract. Therefore, core intestinal microbiota in all humans may vary within a limited range.

In average, Bacteroidetes (60.00%), Firmicutes (29.04%), and Proteobacteria (5.40%) represented 90.00% of sequences identified in Tibetans; this result agrees with those of previous studies, revealing that majority of human gut microbiota can be attributed to these phyla^2,20,23^. F/B ratio (0.48) was low in Tibetans; this result may be related to dietary habits and host physiology^25,26^. These observations agree with those of other studies on Mongolians (0.71)^22^. Both populations consumed considerable amounts of meat (beef and mutton), butter, milk and other dairy products. However, the ratio was much lower than that in Koreans and Westerners. F/B ratios from different locations followed the order GN < HY < LS < TZ < NQ < GC. NQ and GC are pure pastoral areas at high altitudes where the traditional herd-eating habit is maintained. Frequent consumption of meat, dairy products and rare fruits and vegetables possibly explain the higher F/B ratio in these areas than in the other areas. GN, LS and TZ belong to agricultural–pastoral areas where highland barley and cooked wheaten food are staple meals. The relatively high vegetable and fruit intake may lead to a reduced F/B ratio in these places. Interestingly, a low F/B ratio was found for HY, although it is a pure pastoral area at a high elevation. We assume that this condition may be related to the convenient transportation of fruits and vegetables that are imported into this area, which leads to changed meat-eating habits and decreased F/B ratios.

In our study, four CSTs were detected in the Tibetans. Three of these CSTs were also detected in ethnic groups featured in previous enterotype studies^24,27^, namely, *Bacteroides* (enterotype 1), *Prevotella* (enterotype 2) and *Ruminococcus* (enterotype 3). Highest number of samples belonged to *Prevotella*-types, whereas *Bacteroides* contained the second highest amount. *Prevotella* is a SCFA-producing genus, and it is important in maintaining gut homeostasis in high-altitude locations^2,23^. *Prevotella* includes a wide array of carbohydrate- and protein-fermenting and acetate- and H_2_-producingbacteria, whereas *Bacteroides* is associated with metabolism of animal proteins, a variety of amino acids, and saturated fats^18,28^. Traditional Tibetans exhibited high consumption of fried wheaten food, red meat, and fermented dairy products with low quantities of vegetables and fruits; this result was expected as the two genera dominated bacterial composition. High levels of *Prevotella* were typical characteristic of rural populations and agrarian societies^15^. However, urbanization level was the highest in LS, and percentage of *Prevotella* was not the lowest. Further studies are required to determine causes of these results.

Six locations were situated at different altitude levels (from 2000 m to 4000 m). People living in high altitudes featured high bacterial diversity and richness. Clostridiales were signature organisms in samples from locations between 3000 and 4000 m altitudes. *Faecalibacterium* was positive within creasing altitude. Relative abundance of strict anaerobe *Bifidobacterium*, which were sampled from GC, NQ, and LS (altitude above 3000 m), was higher than that from GN and TZ (below 3000 m). Fecal microbiota analysis revealed that at high altitudes, total aerobes decreased significantly with increasing total facultative anaerobes^2^. These groups can produce SCFAs that not only provide energy but also decrease blood pressure via olfactory receptor 78 and G-protein couple receptor 41^29^, benefitting adaptation to energy demands and pulmonary hypertension^30–32^. Metabolites produced by microbiota may play important roles in regulating host health by participating in host metabolism^14^. Microbiota can use non-digestible carbohydrates in the colon and produce SCFAs, namely, acetate, propionate, and butyrate. The results suggest that gut microbiota potentially influence human health by modulating energy harvest and blood pressure response to hypoxic environment at high altitudes. Altitude may play a certain role on gut microbiota based on our results, and it is probably related to the horizontal spread of microbes between individuals, since they might be viable for shorter periods after defecation than they would be in a warm, moist environment.

In conclusion, this study revealed that Tibetans living at high altitudesmanifest low F/B ratios. Significant differences in gut microbiota were observed among different locations, altitudes, and ages. Four CSTs were detected. Gut microbiota play important roles in regulating high-altitude adaptation and high-fat diets.

## Material and Methods

### Study site and sampling

**Figure 7:**
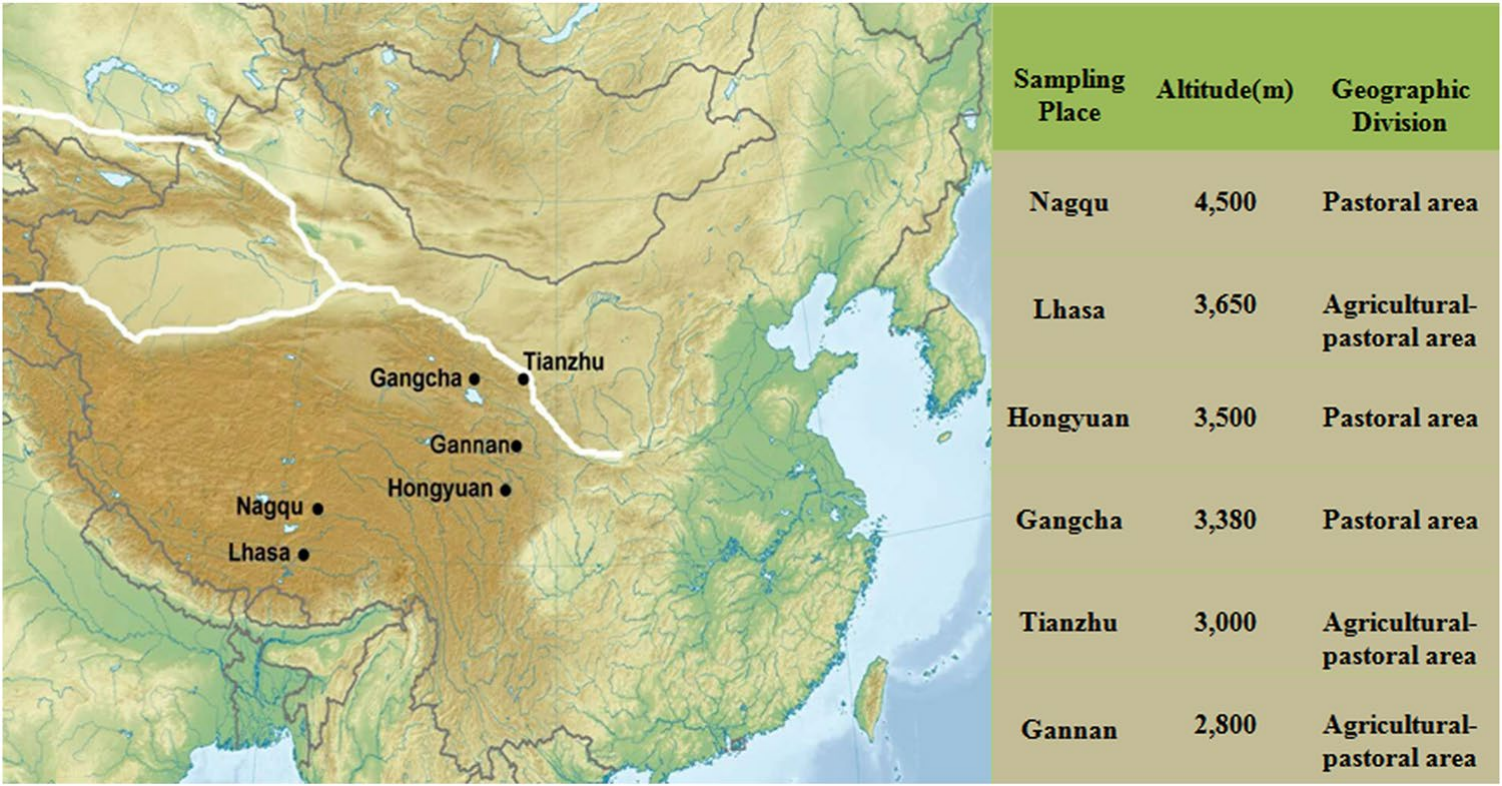
A map of sampling sites. Sampling sites are mapped using MapGIS 10.2 Desktop software (http://www.mapgis.com/index.php/index-view-aid-977.html, Chinese software).

In the present study, 208 healthy Tibetan volunteers were recruited for sampling, which covered six places across the Tibetan Plateau; areas included GC (30 samples), GN (52 samples), HY (29 samples), LS(32 samples), NQ (34 samples), and TZ(30 samples) (Fig. 7). The ages of the participants ranged from 0.7 to 86 years. They were grouped into different age brackets, namely, infant (1–4 years old), children (5–12 years old), juvenile (13–18 years old), youth (19–39 years old), middle (40–59 years old) and old (above 60 years old). Height and weight were available for calculating BMI (Table S2). BMI was classified into four, namely, underweight (BMI < 18.5), normal (18.5 ≤ BMI < 23), overweight (23 ≤ BMI < 25) and obese (BMI ≥ 25), according to the revised Asia–Pacific BMI criteria by the World Health Organization Western Pacific Region.

All volunteers recruited in this study were indigenous residents and resided in the same locality for at least three generations without marrying individuals from any other ethnic groups and never left Tibet. These people did not experience bowel or metabolic diseases nor take any antibiotics or probiotics within three months before sampling dates. Table S2 lists detailed information, e.g., age, gender, and elevation. Sampling was according to methods published previously^2^. Fecal samples were maintained in liquid nitrogen immediately after collection and stored at −80 °C before further experiments. This study was approved by the Ethics Committee of Southwest University for Nationalities, and informed consent was obtained from all volunteers before enrollment in the study. All experiments were performed in accordance with approved guidelines and regulations.

### DNA extraction

Fecal DNA was extracted using QIAampDNA stool minikit according to the instructions of the manufacturer, with a modified pretreatment protocol of the bead-beating procedure described by Schnorr *et al*.^20^. Amount of DNA was determined by Nanodrop ND-2000 (Nanodrop, USA). Purity and quality of genomic DNA were checked on 0.8% agarose gels.

### Polymerase chain reaction (PCR) amplification and high-throughput sequencing

V4 hypervariable region of bacterial 16S rRNA gene was amplified with primers 515F (GTGCCAGCMGCCGCGGTAA) and 806R (GGACTACVSGGGTAT- CTAAT)^33^. For each fecal DNA sample, a 10-digit barcode sequence was added to 5′ ends of forward and reverse primers. PCR was performed on a Mastercycler Gradient (Eppendorf, Germany) using 50 µl reaction volume containing 5 µl10 × Ex Taq buffer (Mg^2+^ plus), 4 µl 12.5 mM dNTPmix (each), 1.25 U Ex Taq DNA polymerase, 2 µl template DNA, 200 nM barcoded primers with 967F and 1406R each, and 36.75 µl ddH_2_O. Cycling parameters were 94 °C for 2 min, followed by 30 cycles at 94 °C for 30 s, 57 °C for 30 s, and 72 °C for 30 s with a final extension at 72 °C for 10 min. Three PCR products per sample were pooled to mitigate reaction-level PCR biases. PCR products were purified using a QIAquick gel extraction kit (QIAGEN, Germany) and quantified using real-time PCR. Amplification product was deep-sequenced using Illumina MiSeq platform at BGI (Shen zhen). After the run, image analysis, base calling, and error estimation were performed using Illumina Analysis Pipeline Version 2.6.

### Data analyses

Raw data were first screened, and sequences were removed from considerations when they spanned less than 200 bp. These data presented low-quality score ≤ 20 and contained ambiguous bases or did not exactly match primer sequences and barcode tags. Qualified reads were separated into different samples using sample-specific barcode sequences and trimmed with Illumina Analysis Pipeline Version 2.6. Next, dataset was analyzed using QIIME^34^. Sequences were clustered into OTUs at a similarity level of 97% to generate rarefaction curves^35^ and to calculate richness and diversity indices^36^. RDP classifier tool^37^ was used to classify all sequences into different taxonomic groups.

Core OTUs presented in all samples were detected using QIIME. Clustering analyses and PCA were used based on OTU information from each sample using R package to examine similarity between different samples. VENN analyses were also conducted using R package. Statistical analyses between different groups were analyzed using ANOVA^38^. Mann–Whitney U test was used for diversity and taxonomic comparisons between groups at different levels (phylum, class, order, family, and genus)^39^. CCA was used to evaluate linkages between gut microbial structure and environmental attributes using R package. LEfSe^40^ was used to detect unique bacterial tax among different groups. To determine the different CSTs across all the locations, hierarchical clustering into CSTs based on genus composition and abundance was conducted according to the methods described by DiGiulio *et al*.^41^.

### Data Availability

The raw sequences of this study have been deposited in the Sequence Read Archive (accession number: SRA551593).

## Electronic supplementary material

Supplementary Information
