# Author Correction: Correlations between gut microbiota community structures of Tibetans and geography

**DOI:** 10.1038/s41598-018-23386-3

**Published:** 2018-03-19

**Authors:** Daoliang Lan, Wenhui Ji, Baoshan Lin, Yabing Chen, Cai Huang, Xianrong Xiong, Mei Fu, Tserang Donko Mipam, Yi Ai, Bo Zeng, Ying Li, Zhixin Cai, Jiangjiang Zhu, Dawei Zhang, Jian Li

**Affiliations:** 1 Institute of Qinghai-Tibetan Plateau, Southwest University for Nationalities, Chengdu, 610041 People’s Republic of China; 2 College of Life Science and Technology, Southwest University for Nationalities, Chengdu, 610041 People’s Republic of China; 3 Animal Disease Prevention and Control Center of Aba Tibetan and Qiang Autonomous Prefecture, Sichuan Province, 624000 People’s Republic of China; 4 Farm Animal Genetic Resources Exploration and Innovation Key Laboratory of Sichuan Province, Sichuan Agricultural University, Chengdu, 611130 People’s Republic of China

Correction to: *Scientific Reports* 10.1038/s41598-017-17194-4, published online 05 December 2017

The original version of this Article contained a typographical error in the spelling of the author Ying Li, which was incorrectly given as Yin Li. This has now been corrected in the PDF and HTML versions of the Article, and in the accompanying Supplementary Information File.

