# Supplementary material for: Correlations between gut microbiota community structures of Tibetans and geography
Source: Sci Rep. 2017 Dec 5;7:16982. doi: 10.1038/s41598-017-17194-4 (PMC5717229; doi:10.1038/s41598-017-17194-4)
Supplement: Supplementary file 1 — Supplementary Information [file 41598_2017_17194_MOESM1_ESM.doc]

Title: Correlations between gut microbiota community structures of Tibetans and geography

Authors: Daoliang Lan^1^,^2*^, Wenhui Ji^2^, Baoshan Lin^2,3^, Yabing Chen^2^, Cai Huang^2^, Xianrong Xiong^2^, Mei Fu^2^, Tserang Donko Mipam^1^,Yi Ai^1^, Bo Zeng^4^, Ying Li^4^, Zhixin Cai^1^, Jiangjiang Zhu^1^, Dawei Zhang^1^, JianLi^1,2*^

Address: ^1^Institute of Qinghai-Tibetan Plateau, Southwest University for Nationalities, Chengdu, 610041, Peoples’ Republic of China.

^2^College of Life Science and Technology, Southwest University for Nationalities, Chengdu ,610041, Peoples’ Republic of China.

^3^Animal Disease Prevention and Control Center of Aba Tibetan and Qiang Autonomous Prefecture, Sichuan Province,624000, Peoples’ Republic of China.

^4^Farm Animal Genetic Resources Exploration and Innovation Key Laboratory of Sichuan Province, Sichuan Agricultural University, Chengdu, 611130, Peoples’ Republic of China.

**Table S1. The statistical information of sequencing in each sample.**

| **Samples** | **No. of sequences** | **No. of OTUs** | **Samples** | **No. of sequences** | **No. of OTUs** |
| --- | --- | --- | --- | --- | --- |
| A51 | 16455 | 284 | B78 | 70314 | 332 |
| A52 | 105450 | 226 | B79 | 77331 | 198 |
| A53 | 81009 | 160 | B80 | 78098 | 271 |
| A54 | 75827 | 276 | B81 | 90631 | 363 |
| A55 | 73216 | 336 | B82 | 88672 | 322 |
| A56 | 84000 | 362 | B83 | 92758 | 282 |
| A123 | 96589 | 222 | B92 | 66786 | 348 |
| A121 | 57645 | 309 | B93 | 70527 | 444 |
| A126 | 31536 | 191 | A38 | 60550 | 540 |
| A124 | 12594 | 190 | A39 | 81570 | 500 |
| A122 | 88468 | 230 | A40 | 22131 | 413 |
| A125 | 63417 | 287 | A41 | 85265 | 395 |
| A127 | 73120 | 190 | A42 | 78802 | 284 |
| A119 | 76573 | 191 | A43 | 92486 | 427 |
| A120 | 69423 | 392 | A44 | 102357 | 461 |
| A158 | 61356 | 327 | A45 | 54369 | 250 |
| B31 | 95247 | 211 | A46 | 71701 | 405 |
| B32 | 70302 | 352 | A47 | 112772 | 190 |
| B33 | 64963 | 324 | A48 | 84754 | 231 |
| B34 | 67781 | 161 | A49 | 36874 | 191 |
| B35 | 84745 | 320 | A50 | 109189 | 365 |
| B36 | 73618 | 320 | B55 | 78035 | 161 |
| B37 | 82350 | 201 | B56 | 67250 | 322 |
| B38 | 93863 | 355 | B57 | 79597 | 236 |
| B39 | 89682 | 204 | B58 | 89909 | 371 |
| B40 | 70232 | 285 | B59 | 65991 | 308 |
| B85 | 70525 | 326 | B60 | 91082 | 377 |
| B86 | 65985 | 232 | B61 | 64384 | 286 |
| B87 | 87749 | 383 | B62 | 75064 | 339 |
| B88 | 86546 | 426 | B63 | 95327 | 199 |
| A80 | 89352 | 369 | B64 | 71713 | 234 |
| A82 | 115554 | 341 | B65 | 93830 | 387 |
| A83 | 50994 | 126 | B66 | 83293 | 313 |
| A84 | 53511 | 176 | B67 | 86047 | 332 |
| A86 | 84654 | 386 | B68 | 98738 | 410 |
| A87 | 72506 | 412 | B69 | 75001 | 380 |
| A88 | 85590 | 302 | B70 | 93211 | 373 |
| A90 | 95798 | 252 | B71 | 89329 | 385 |
| A91 | 82259 | 166 | B94 | 67565 | 355 |
| A93 | 80526 | 290 | B95 | 71077 | 283 |
| A94 | 69614 | 512 | A18 | 67209 | 466 |
| A95 | 113368 | 319 | A19 | 65598 | 460 |
| A96 | 99965 | 211 | A20 | 94179 | 310 |
| A97 | 105867 | 150 | A21 | 65717 | 379 |
| A98 | 90089 | 429 | A22 | 96428 | 519 |
| A101 | 75116 | 432 | A23 | 88628 | 435 |
| A103 | 82756 | 415 | A24 | 100001 | 229 |
| A106 | 76078 | 196 | A25 | 60624 | 429 |
| A107 | 60630 | 309 | A26 | 61443 | 377 |
| A108 | 87636 | 331 | A27 | 76501 | 187 |
| A110 | 75813 | 341 | A28 | 76391 | 394 |
| A111 | 79610 | 253 | A29 | 65065 | 352 |
| A112 | 98415 | 261 | A30 | 71769 | 204 |
| A113 | 58738 | 447 | A31 | 72220 | 410 |
| A114 | 99596 | 244 | A32 | 114855 | 194 |
| A115 | 81080 | 515 | A33 | 62026 | 348 |
| A116 | 111071 | 239 | A34 | 61554 | 526 |
| A128 | 74273 | 250 | A35 | 87213 | 212 |
| A129 | 67834 | 301 | A36 | 68457 | 515 |
| A130 | 106535 | 261 | A37 | 68028 | 403 |
| A132 | 91804 | 424 | B41 | 88215 | 464 |
| A138 | 83469 | 233 | B42 | 60683 | 426 |
| A141 | 98241 | 256 | B43 | 86386 | 411 |
| A142 | 97263 | 249 | B44 | 78066 | 365 |
| A143 | 69636 | 385 | B45 | 86470 | 378 |
| A144 | 25626 | 166 | B46 | 76986 | 132 |
| A146 | 125895 | 222 | B47 | 78957 | 365 |
| A147 | 94367 | 160 | B48 | 74867 | 313 |
| A148 | 87519 | 360 | B49 | 84901 | 434 |
| A149 | 20959 | 123 | B50 | 85203 | 497 |
| A150 | 108674 | 264 | B51 | 72424 | 344 |
| A151 | 88778 | 223 | B52 | 76664 | 345 |
| A152 | 102441 | 295 | B53 | 89476 | 335 |
| A153 | 99507 | 167 | B54 | 89173 | 278 |
| A154 | 71816 | 300 | A63 | 89560 | 161 |
| A155 | 113978 | 244 | A64 | 99156 | 173 |
| A156 | 78979 | 214 | A65 | 86915 | 152 |
| A157 | 118985 | 232 | A66 | 46441 | 266 |
| A159 | 76066 | 188 | A67 | 73784 | 208 |
| A160 | 91565 | 253 | A68 | 87723 | 188 |
| A161 | 62823 | 370 | A145 | 88508 | 218 |
| A162 | 68286 | 99 | B1 | 65834 | 337 |
| A1 | 78897 | 440 | B2 | 87536 | 184 |
| A2 | 99663 | 227 | B3 | 93563 | 133 |
| A3 | 75936 | 283 | B4 | 74321 | 260 |
| A4 | 68346 | 267 | B5 | 99802 | 188 |
| A5 | 43582 | 100 | B6 | 97773 | 281 |
| A6 | 32936 | 324 | B7 | 88384 | 334 |
| A7 | 82946 | 282 | B8 | 65939 | 204 |
| A8 | 65376 | 203 | B10 | 80976 | 234 |
| A9 | 109472 | 158 | B11 | 74442 | 201 |
| A10 | 64456 | 380 | B12 | 102115 | 239 |
| A11 | 34415 | 186 | B13 | 69015 | 294 |
| A12 | 110933 | 210 | B14 | 85326 | 214 |
| A13 | 70011 | 159 | B15 | 79696 | 285 |
| A15 | 96217 | 226 | B16 | 80955 | 277 |
| A16 | 84085 | 425 | B17 | 96223 | 155 |
| A17 | 76322 | 418 | B18 | 84233 | 283 |
| B72 | 68770 | 459 | B19 | 82889 | 222 |
| B73 | 64738 | 398 | B20 | 72897 | 260 |
| B74 | 82017 | 485 | B84 | 98217 | 326 |
| B75 | 74681 | 351 | B89 | 81936 | 207 |
| B76 | 64391 | 379 | B90 | 67314 | 321 |
| B77 | 90555 | 467 | B91 | 80639 | 212 |


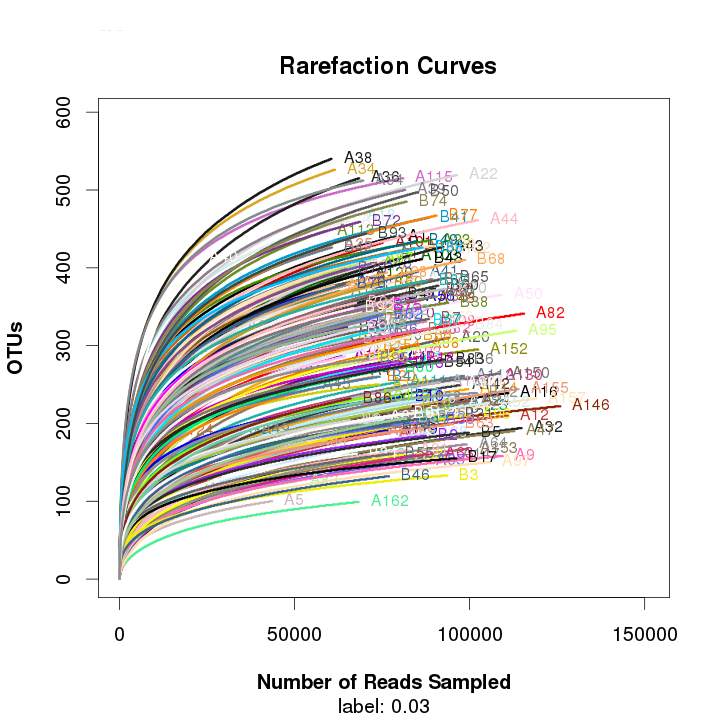


**Figure S1. Rarefaction curves of each sample.**


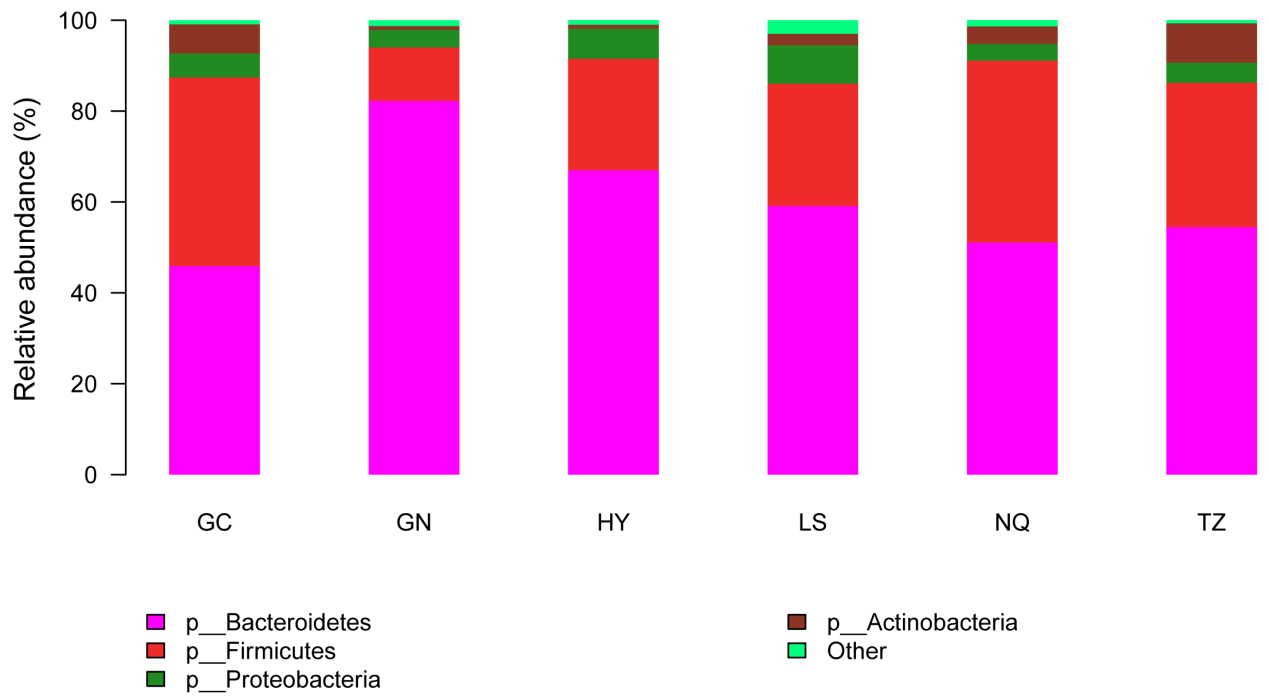


**Figure S2. Taxonomic classification of bacterial reads at the phylum level.** Sequences were classified into different taxonomic groups using Ribosomal Database Project (RDP) classifier with a threshold value of 80%.

**
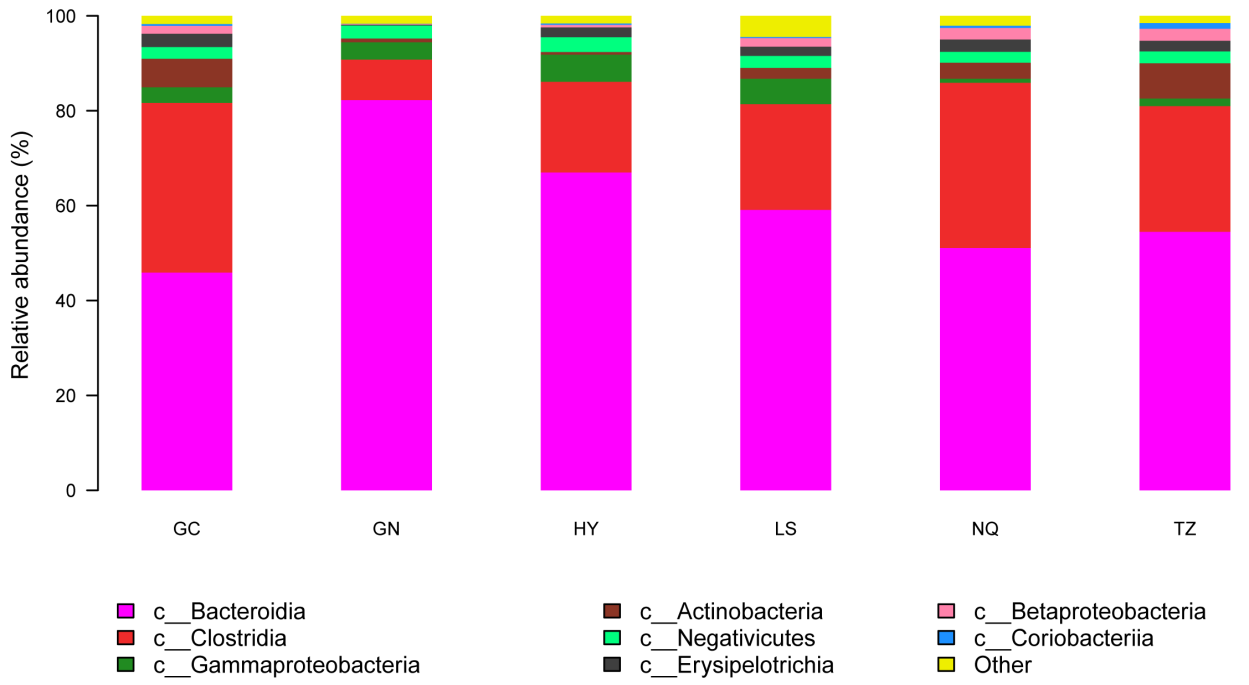
**

**Figure S3. Taxonomic classification of bacterial reads at the class level.** Sequences were classified into different taxonomic groups using RDP classifier with a threshold value of 80%.

**
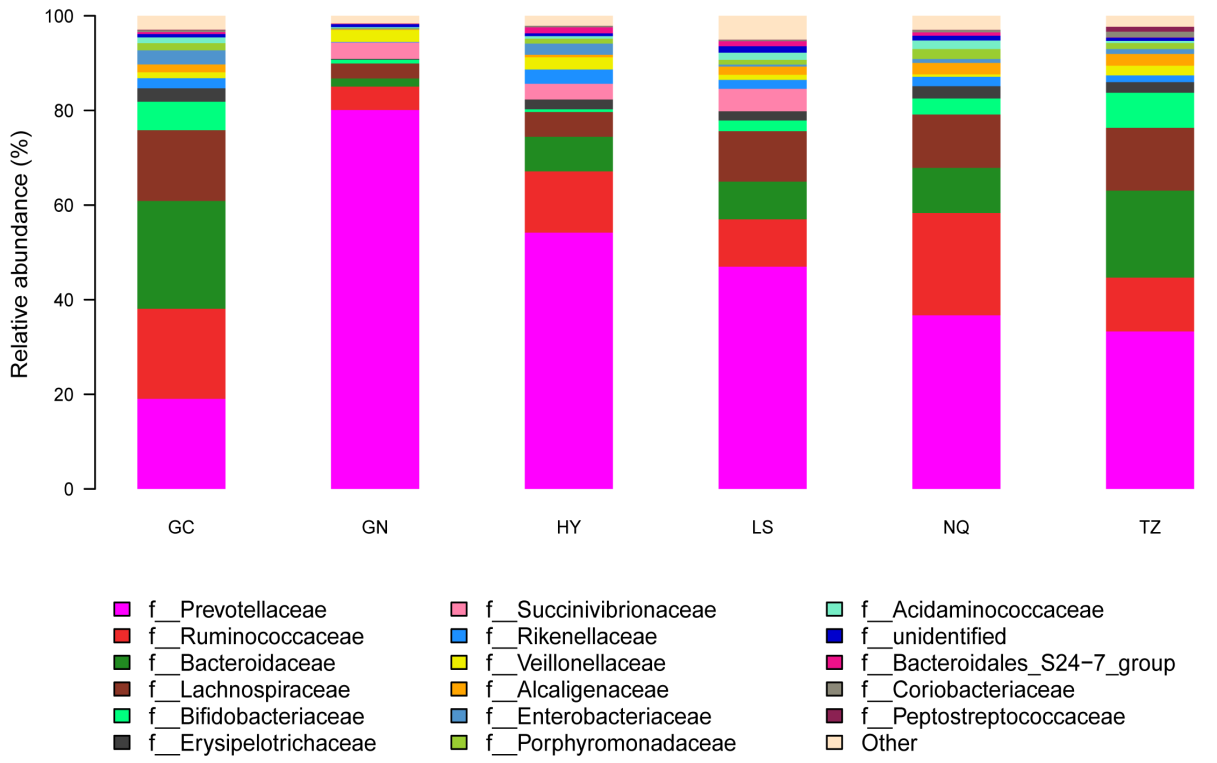
**

**Figure S4. Taxonomic classification of bacterial reads at the family level.** Sequences were classified into different taxonomic groups using RDP classifier with a threshold value of 80%.

**
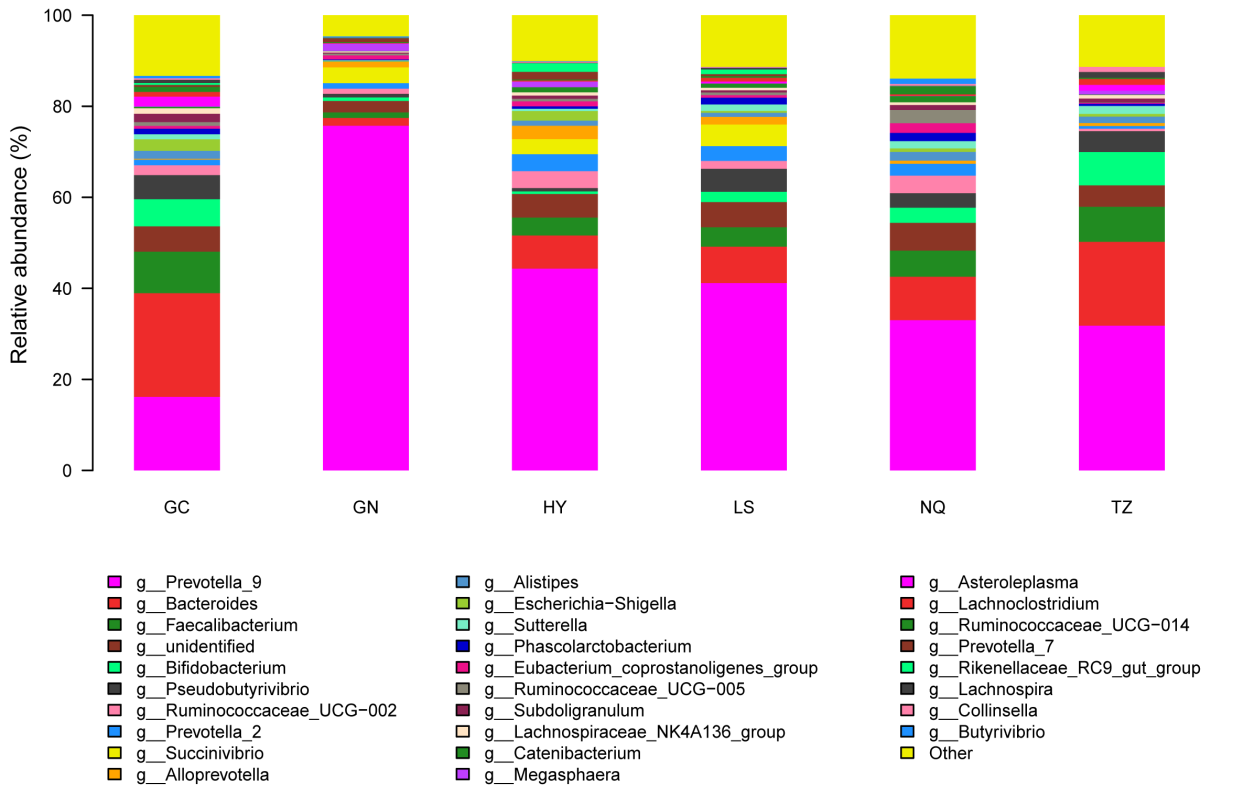
**

**Figure S5. Taxonomic classification of bacterial reads at the genus level.** Sequences were classified into different taxonomic groups using RDP classifier with a threshold value of 80%.

**Table S2. Sample Information.**

| **Sample ID** | **Location** | **Altitude (m)** | **Sex** | **Age (years)** | **Height (cm)** | **Weight(kg)** |
| --- | --- | --- | --- | --- | --- | --- |
| A1 | Hongyuan | 3500 | female | 50 | 156 | 50 |
| A2 | Hongyuan | 3500 | female | 21 | 156 | 58 |
| A3 | Hongyuan | 3500 | male | 7 | 167 | 60 |
| A4 | Hongyuan | 3500 | male | 69 | 176 | 59 |
| A5 | Hongyuan | 3500 | male | 39 | 176 | 60 |
| A6 | Hongyuan | 3500 | female | 65 | 150 | 45 |
| A7 | Hongyuan | 3500 | female | 14 | 146 | 54 |
| A8 | Hongyuan | 3500 | female | 24 | 156 | 55 |
| A9 | Hongyuan | 3500 | female | 26 | 153 | 42 |
| A10 | Hongyuan | 3500 | female | 79 | 156 | 58 |
| A11 | Hongyuan | 3500 | female | 23 | 157 | 50 |
| A12 | Hongyuan | 3500 | female | 61 | 154 | 40 |
| A13 | Hongyuan | 3500 | male | 4 | 85 | 18 |
| A15 | Hongyuan | 3500 | female | 58 | 155 | 60 |
| A16 | Hongyuan | 3500 | male | 4 | 126 | 16 |
| A17 | Hongyuan | 3500 | female | 22 | 152 | 42 |
| B72 | Hongyuan | 3500 | male | 72 | 170 | 50 |
| B73 | Hongyuan | 3500 | male | 77 | 160 | 70 |
| B74 | Hongyuan | 3500 | female | 24 | 153 | 40 |
| B75 | Hongyuan | 3500 | female | 14 | 152 | 35 |
| B76 | Hongyuan | 3500 | female | 52 | 159 | 62 |
| B77 | Hongyuan | 3500 | female | 67 | 154 | 60 |
| B78 | Hongyuan | 3500 | male | 80 | 162 | 75 |
| B79 | Hongyuan | 3500 | male | 13 | 156 | 42 |
| B80 | Hongyuan | 3500 | female | 5 | 95 | 25 |
| B81 | Hongyuan | 3500 | female | 44 | 170 | 50 |
| B82 | Hongyuan | 3500 | male | 69 | 164 | 50 |
| B83 | Hongyuan | 3500 | female | 15 | 156 | 50 |
| B92 | Hongyuan | 3500 | female | 49 | 168 | 65 |
| B93 | Hongyuan | 3500 | female | 15 | 152 | 60 |
| A18 | Nagqu | 4500 | male | 0.7 | 40 | 5 |
| A19 | Nagqu | 4500 | female | 42 | 150 | 45 |
| A20 | Nagqu | 4500 | male | 58 | 178 | 82 |
| A21 | Nagqu | 4500 | male | 32 | 175 | 60 |
| A22 | Nagqu | 4500 | male | 67 | 160 | 50 |
| A23 | Nagqu | 4500 | female | 32 | 160 | 49 |
| A24 | Nagqu | 4500 | female | 3 | 90 | 13 |
| A25 | Nagqu | 4500 | female | 71 | 168 | 50 |
| A26 | Nagqu | 4500 | female | 4 | 90 | 12 |
| A27 | Nagqu | 4500 | female | 38 | 158 | 49 |
| A28 | Nagqu | 4500 | male | 43 | 165 | 61 |
| A29 | Nagqu | 4500 | male | 33 | 166 | 51 |
| A30 | Nagqu | 4500 | female | 24 | 155 | 48 |
| A31 | Nagqu | 4500 | female | 86 | 160 | 45 |
| A32 | Nagqu | 4500 | male | 35 | 172 | 60 |
| A33 | Nagqu | 4500 | male | 26 | 165 | 52 |
| A34 | Nagqu | 4500 | female | 65 | 161 | 48 |
| A35 | Nagqu | 4500 | male | 56 | 160 | 49 |
| A36 | Nagqu | 4500 | male | 47 | 175 | 54 |
| A37 | Nagqu | 4500 | female | 83 | 165 | 49 |
| B41 | Nagqu | 4500 | female | 32 | 165 | 49 |
| B42 | Nagqu | 4500 | male | 40 | 165 | 52 |
| B43 | Nagqu | 4500 | male | 30 | 166 | 50 |
| B44 | Nagqu | 4500 | male | 45 | 160 | 48 |
| B45 | Nagqu | 4500 | male | 60 | 156 | 50 |
| B46 | Nagqu | 4500 | male | 28 | 172 | 76 |
| B47 | Nagqu | 4500 | male | 4 | 92 | 16 |
| B48 | Nagqu | 4500 | female | 35 | 160 | 52 |
| B49 | Nagqu | 4500 | male | 30 | 150 | 54 |
| B50 | Nagqu | 4500 | female | 4 | 90 | 15 |
| B51 | Nagqu | 4500 | female | 34 | 150 | 48 |
| B52 | Nagqu | 4500 | male | 40 | 170 | 65 |
| B53 | Nagqu | 4500 | male | 26 | 168 | 52 |
| B54 | Nagqu | 4500 | female | 3 | 86 | 15 |
| A38 | Lhasa | 3650 | female | 17 | 160 | 56 |
| A39 | Lhasa | 3650 | male | 55 | 168 | 55 |
| A40 | Lhasa | 3650 | male | 81 | 160 | 50 |
| A41 | Lhasa | 3650 | female | 60 | 158 | 52 |
| A42 | Lhasa | 3650 | female | 4 | 85 | 25 |
| A43 | Lhasa | 3650 | female | 28 | 156 | 50 |
| A44 | Lhasa | 3650 | female | 54 | 150 | 62 |
| A45 | Lhasa | 3650 | male | 64 | 180 | 70 |
| A46 | Lhasa | 3650 | female | 21 | 160 | 55 |
| A47 | Lhasa | 3650 | female | 45 | 152 | 51 |
| A48 | Lhasa | 3650 | female | 58 | 149 | 53 |
| A49 | Lhasa | 3650 | female | 2 | 55 | 15 |
| A50 | Lhasa | 3650 | male | 72 | 168 | 65 |
| B55 | Lhasa | 3650 | female | 22 | 157 | 52 |
| B56 | Lhasa | 3650 | male | 23 | 172 | 58 |
| B57 | Lhasa | 3650 | male | 28 | 170 | 65 |
| B58 | Lhasa | 3650 | male | 22 | 171 | 53 |
| B59 | Lhasa | 3650 | female | 23 | 165 | 48 |
| B60 | Lhasa | 3650 | female | 23 | 160 | 53 |
| B61 | Lhasa | 3650 | female | 9 | 149 | 39 |
| B62 | Lhasa | 3650 | female | 29 | 156 | 47 |
| B63 | Lhasa | 3650 | female | 57 | 155 | 55 |
| B64 | Lhasa | 3650 | female | 23 | 155 | 50 |
| B65 | Lhasa | 3650 | male | 26 | 170 | 60 |
| B66 | Lhasa | 3650 | female | 22 | 158 | 50 |
| B67 | Lhasa | 3650 | male | 19 | 170 | 51 |
| B68 | Lhasa | 3650 | female | 55 | 154 | 57 |
| B69 | Lhasa | 3650 | female | 24 | 155 | 50 |
| B70 | Lhasa | 3650 | male | 25 | 169 | 60 |
| B71 | Lhasa | 3650 | male | 28 | 168 | 58 |
| B94 | Lhasa | 3650 | male | 30 | 173 | 63 |
| B95 | Lhasa | 3650 | male | 22 | 171 | 75 |
| A51 | Gangcha | 3380 | female | 85 | 140 | 53 |
| A52 | Gangcha | 3380 | male | 19 | 170 | 61 |
| A53 | Gangcha | 3380 | male | 17 | 172 | 60 |
| A54 | Gangcha | 3380 | female | 23 | 161 | 55 |
| A55 | Gangcha | 3380 | female | 5 | 112 | 20 |
| A56 | Gangcha | 3380 | male | 19 | 169 | 67 |
| A123 | Gangcha | 3380 | female | 21 | 159 | 65 |
| A121 | Gangcha | 3380 | female | 17 | 164 | 58 |
| A126 | Gangcha | 3380 | female | 42 | 161 | 70 |
| A124 | Gangcha | 3380 | female | 15 | 161 | 55 |
| A122 | Gangcha | 3380 | male | 35 | 175 | 91 |
| A125 | Gangcha | 3380 | male | 46 | 172 | 65 |
| A127 | Gangcha | 3380 | female | 43 | 160 | 60 |
| A119 | Gangcha | 3380 | male | 22 | 169 | 61 |
| A120 | Gangcha | 3380 | female | 46 | 158 | 62 |
| A158 | Gangcha | 3380 | female | 42 | 156 | 60 |
| B31 | Gangcha | 3380 | female | 49 | 150 | 63 |
| B32 | Gangcha | 3380 | male | 47 | 179 | 65 |
| B33 | Gangcha | 3380 | male | 51 | 173 | 80 |
| B34 | Gangcha | 3380 | female | 44 | 167 | 62 |
| B35 | Gangcha | 3380 | male | 44 | 173 | 80 |
| B36 | Gangcha | 3380 | female | 42 | 152 | 45 |
| B37 | Gangcha | 3380 | male | 37 | 176 | 73 |
| B38 | Gangcha | 3380 | female | 38 | 165 | 53 |
| B39 | Gangcha | 3380 | male | 43 | 178 | 65 |
| B40 | Gangcha | 3380 | male | 71 | 170 | 55 |
| B85 | Gangcha | 3380 | female | 44 | 160 | 55 |
| B86 | Gangcha | 3380 | female | 39 | 156 | 54 |
| B87 | Gangcha | 3380 | male | 64 | 162 | 60 |
| B88 | Gangcha | 3380 | female | 42 | 165 | 62 |
| A63 | Tianzhu | 3000 | male | 42 | 167 | 66 |
| A64 | Tianzhu | 3000 | male | 49 | 165 | 73 |
| A65 | Tianzhu | 3000 | female | 41 | 160 | 55 |
| A66 | Tianzhu | 3000 | female | 51 | 150 | 69 |
| A67 | Tianzhu | 3000 | male | 42 | 170 | 72 |
| A68 | Tianzhu | 3000 | female | 42 | 155 | 68 |
| A145 | Tianzhu | 3000 | female | 49 | 157 | 71 |
| B1 | Tianzhu | 3000 | female | 49 | 155 | 50 |
| B2 | Tianzhu | 3000 | female | 52 | 160 | 57 |
| B3 | Tianzhu | 3000 | male | 51 | 162 | 60 |
| B4 | Tianzhu | 3000 | female | 46 | 161 | 50 |
| B5 | Tianzhu | 3000 | female | 31 | 160 | 52 |
| B6 | Tianzhu | 3000 | male | 53 | 170 | 82 |
| B7 | Tianzhu | 3000 | male | 2.5 | 80 | 15 |
| B8 | Tianzhu | 3000 | male | 55 | 165 | 69 |
| B10 | Tianzhu | 3000 | female | 50 | 165 | 60 |
| B11 | Tianzhu | 3000 | male | 27 | 178 | 75 |
| B12 | Tianzhu | 3000 | male | 38 | 163 | 67 |
| B13 | Tianzhu | 3000 | male | 72 | 146 | 42 |
| B14 | Tianzhu | 3000 | female | 40 | 150 | 50 |
| B15 | Tianzhu | 3000 | male | 13 | 135 | 35 |
| B16 | Tianzhu | 3000 | female | 23 | 160 | 50 |
| B17 | Tianzhu | 3000 | female | 49 | 160 | 60 |
| B18 | Tianzhu | 3000 | male | 52 | 170 | 60 |
| B19 | Tianzhu | 3000 | female | 52 | 168 | 60 |
| B20 | Tianzhu | 3000 | male | 10 | 110 | 30 |
| B84 | Tianzhu | 3000 | male | 50 | 165 | 72 |
| B89 | Tianzhu | 3000 | female | 42 | 158 | 55 |
| B90 | Tianzhu | 3000 | female | 73 | 155 | 50 |
| B91 | Tianzhu | 3000 | female | 43 | 161 | 60 |
| A80 | Gannan | 2800 | female | 40 | 155 | 48 |
| A82 | Gannan | 2800 | female | 32 | 158 | 63 |
| A83 | Gannan | 2800 | female | 70 | 148 | 39 |
| A84 | Gannan | 2800 | male | 3 | 80 | 11 |
| A86 | Gannan | 2800 | female | 46 | 161 | 58 |
| A87 | Gannan | 2800 | male | 61 | 171 | 68 |
| A88 | Gannan | 2800 | female | 30 | 154 | 59 |
| A90 | Gannan | 2800 | male | 6 | 107 | 19 |
| A91 | Gannan | 2800 | male | 5 | 102 | 16 |
| A93 | Gannan | 2800 | male | 7 | 107 | 21 |
| A94 | Gannan | 2800 | female | 66 | 148 | 42 |
| A95 | Gannan | 2800 | female | 75 | 141 | 52 |
| A96 | Gannan | 2800 | male | 44 | 176 | 71 |
| A97 | Gannan | 2800 | female | 43 | 150 | 64 |
| A98 | Gannan | 2800 | male | 4 | 93 | 14 |
| A101 | Gannan | 2800 | female | 43 | 158 | 58 |
| A103 | Gannan | 2800 | male | 67 | 165 | 63 |
| A106 | Gannan | 2800 | male | 5 | 98 | 16 |
| A107 | Gannan | 2800 | female | 24 | 157 | 49 |
| A108 | Gannan | 2800 | male | 4 | 85 | 15 |
| A110 | Gannan | 2800 | male | 5 | 100 | 15 |
| A111 | Gannan | 2800 | male | 5 | 105 | 17 |
| A112 | Gannan | 2800 | female | 19 | 152 | 57 |
| A113 | Gannan | 2800 | male | 48 | 170 | 67 |
| A114 | Gannan | 2800 | female | 57 | 155 | 73 |
| A115 | Gannan | 2800 | female | 52 | 146 | 44 |
| A116 | Gannan | 2800 | male | 5 | 95 | 13 |
| A128 | Gannan | 2800 | female | 44 | 160 | 70 |
| A129 | Gannan | 2800 | female | 3 | 88 | 15 |
| A130 | Gannan | 2800 | female | 7 | 110 | 18 |
| A132 | Gannan | 2800 | female | 73 | 153 | 54 |
| A138 | Gannan | 2800 | male | 4 | 105 | 18 |
| A141 | Gannan | 2800 | male | 44 | 175 | 80 |
| A142 | Gannan | 2800 | male | 66 | 158 | 61 |
| A143 | Gannan | 2800 | female | 20 | 156 | 45 |
| A144 | Gannan | 2800 | male | 16 | 165 | 50 |
| A146 | Gannan | 2800 | male | 52 | 175 | 76 |
| A147 | Gannan | 2800 | female | 6 | 116 | 18 |
| A148 | Gannan | 2800 | female | 41 | 162 | 42 |
| A149 | Gannan | 2800 | female | 59 | 153 | 48 |
| A150 | Gannan | 2800 | male | 23 | 169 | 56 |
| A151 | Gannan | 2800 | male | 51 | 176 | 53 |
| A152 | Gannan | 2800 | female | 52 | 162 | 62 |
| A153 | Gannan | 2800 | male | 50 | 159 | 67 |
| A154 | Gannan | 2800 | female | 45 | 155 | 53 |
| A155 | Gannan | 2800 | female | 48 | 145 | 38 |
| A156 | Gannan | 2800 | male | 3 | 65 | 11 |
| A157 | Gannan | 2800 | female | 23 | 163 | 60 |
| A159 | Gannan | 2800 | female | 3 | 75 | 10 |
| A160 | Gannan | 2800 | male | 45 | 167 | 64 |
| A161 | Gannan | 2800 | male | 22 | 170 | 60 |
| A162 | Gannan | 2800 | male | 40 | 165 | 55 |
